# Contemporary bicruciate-retaining total knee arthroplasty implants demonstrate favorable survivorship: a systematic review and meta-analysis of 1576 knees

**DOI:** 10.1007/s00590-026-04811-0

**Published:** 2026-07-01

**Authors:** Varun Kompala, Khaled A. Elmenawi, Maile Wedgwood, Islam A. Sherif, Loren Hackett, Michael S. Ramos, Adolph V. Lombardi, Nicolas S. Piuzzi

**Affiliations:** 1 Department of Orthopaedic Surgery, Cleveland Clinic, Cleveland, OH USA; 2 Floyd D. Loop Alumni Library, Cleveland Clinic, Cleveland, OH USA; 3 Joint Implant Surgeons, Inc., New Albany, OH USA; 4 Department of Biomedical Engineering, Cleveland Clinic, Cleveland, OH USA

**Keywords:** TKA, Total knee arthroplasty, Bicruciate, Biomechanics, Survivorship

## Abstract

**Introduction:**

Bicruciate-retaining (BCR) total knee arthroplasty (TKA) was developed to better replicate native knee biomechanics by preserving both cruciate ligaments. First-generation BCR implants were notorious for technical challenges and suboptimal survivorship. However, advancements in implant design and surgical techniques have renewed interest in second-generation BCR TKA systems. This study aimed to evaluate the overall survivorship of contemporary (second-generation) BCR primary TKA implants.

**Methods:**

A systematic review of PubMed, Scopus, Embase, Web of Science, and Cochrane databases was conducted from inception to January 3, 2025. Inclusion criteria were studies that reported the number of revisions following second-generation BCR TKA. We excluded case reports, review articles, and studies that evaluated first-generation BCR TKA. A total of 1046 articles were retrieved; ultimately, 13 were included. Events per person-years pooled analysis was performed to estimate the incidence of all-cause revision, adjusting for duration of follow-up. Heterogeneity was measured using I^2^ test. A* p*-value < 0.05 was considered statistically significant.

**Results:**

A total of 1,087 BCR TKA implants among 13 studies were analyzed. The mean follow-up was 2.6 years. A total of 62 (5.7%) knees were revised. The overall pooled rate of all-cause revision was 1.6 per 100 person-years (95% confidence interval [CI] 0.009–0.023) Heterogeneity among the analyzed studies was significant (I^2^ = 75.5%, *p* < 0.001).

**Conclusion:**

Contemporary BCR TKA implants showed improved survivorship compared to historical reports, with a low pooled all-cause revision rate of 1.6 per 100 person-years, corresponding to a 1.6% chance of revision per year of follow-up. Despite the associated heterogeneity, these findings suggest that modern BCR designs offer durable outcomes and support their continued use. Further long-term comparative data are needed to better define their role relative to modern knee implants.

**Supplementary Information:**

The online version contains supplementary material available at 10.1007/s00590-026-04811-0.

## Introduction

Total knee arthroplasty (TKA) is one of the most common elective procedures worldwide. U.S. Medicare-based models project that annual primary TKA volume will rise from just over 1 million cases in 2022 to nearly 2.8 million by 2040, a 175% increase in less than two decades [1]. Despite modern advances in implant design, perioperative care, and surgical techniques, up to 19% of patients remain dissatisfied with their postoperative outcome [2]. Consistent with this figure, a large analysis of more than 5,000 primary TKAs further highlights this gap by showing that 16.3% still deemed their knee unacceptable at one year [3].

Many advocate for bicruciate-retaining (BCR) TKA implants due to their ability to preserve both the ACL and posterior cruciate ligament (PCL) to better reproduce native knee kinematics. First-generation BCR systems, introduced in the 1970–1980 s, suffered high tibial loosening and revision rates approaching 15% within the first decade, prompting the concept to be shelved for nearly two decades [4, 5]. Second-generation BCR designs address those shortcomings with anatomically contoured femoral components, asymmetric polyethylene inserts that respect ACL clearance, and highly porous fixation surfaces for better tibial ingrowth. Mid-term results are promising, with five-year survivorship of 96% in a randomized comparison with posterior-stabilized implants [6], and 95% at a mean of five years in a multicenter cohort [7]. Whether such mid-term success translates into long-term durability comparable to mainstream designs remains uncertain.

Therefore, the purpose of our study was to perform a systematic review and meta-analysis of second-generation BCR TKAs. Our objectives were to (i) quantify a pooled revision rate, and (ii) characterize failure modes and their temporal distribution.

## Methods

### Search strategy

The review protocol was drafted prospectively in accordance with the Preferred Reporting Items for Systematic Reviews and Meta-Analyses (PRISMA) 2020 statement [8] and registered to the International Prospective Register of Systematic Reviews (PROSPERO). No protocol deviations occurred after data extraction began. The search was conducted by a healthcare librarian across five databases—CENTRAL (Cochrane Library), Embase (Ovid), MEDLINE (Ovid), Scopus, and Web of Science Core Collection—from their inception through 3 January 2025. Each strategy linked controlled vocabulary (e.g., *Arthroplasty*,* Replacement*,* Knee* in MEDLINE; *knee replacement*/exp in Embase) and free-text terms for knee arthroplasty. The full search strategy is available in **Online Appendix**. Searches were restricted to publications from 2010 onward to capture second-generation designs. No language limits were applied. Duplicate records were removed automatically before screening.

### Inclusion/exclusion criteria

We included adults (≥ 18 y) undergoing primary TKA using second-generation BCR TKA with numbers of all-cause revisions available. We excluded first-generation BCR implants, unicompartmental or non-BCR TKAs, case series with < 10 knees, reviews, conference abstracts without full text, and cadaveric or biomechanical studies. Two reviewers screened independently; disagreements were resolved by consensus with a third author.

**Fig. 1:**
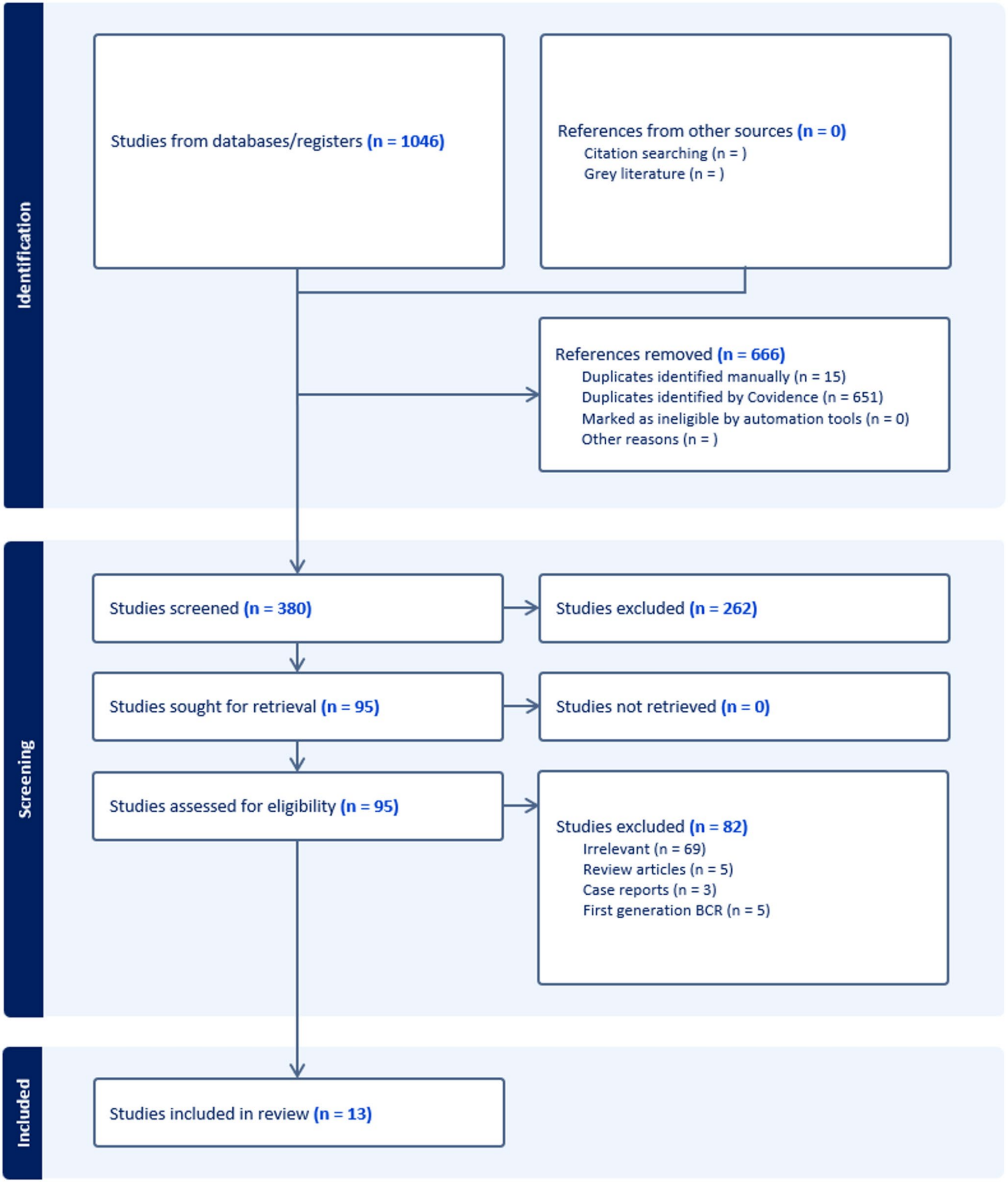
PRISMA diagram

The search yielded 1,046 unique records. After title- and abstract-level screening, 95 full-text articles were assessed, and 13 met all eligibility criteria (Fig. 1). Together, these studies contributed 1,087 second-generation BCR TKAs with a weighted mean follow-up of 2.6 years. Six cohorts evaluated the Vanguard XP design, five the Journey II XR, one the Hermes 2 C, and one did not specify the implant manufacturer.

### Data extraction

Two independent authors collected the data. If available, data collected included study’s author, year, implant system name, number of BCR knees implanted, number of revisions, reported failure mechanisms, and follow-up duration.

### Quality assessment

Study quality was appraised with two complementary tools. Randomized controlled trials were evaluated with the Cochrane Risk-of-Bias 2.0 tool (parallel-group version, 2019), and the remaining 11 observational studies with the Newcastle–Ottawa Scale (NOS) [9]. Detailed domain-level results are presented in the **Online Appendix.**

### Data analyses

Analyses were performed using Comprehensive Meta-analysis Software, under a random effect model to account for differences among analyzed studies. The number of revisions was used to calculate pooled revision rate using per person-years analysis to account for follow-up duration. Heterogeneity was measured using I^2^ test, where I^2^ >50% represents substantial heterogeneity. A P value < 0.05 was considered statistically significant.

## Results

### Study characteristics

Thirteen studies met all eligibility criteria [6, 7, 10–20], contributing 1,087 s-generation BCR TKAs (Table 1). Mean follow-up was 2.6 years. Implant families represented were Vanguard XP (6 studies), Journey II XR (5 studies), Hermes 2 C (1 study), and “unspecified BCR” (1 studies).

**Table 1:** Summary of Included Studies

Study	Study design	BCR implant name	No. BCR Patients	No. BCR Revised	Causes for Revision	Duration of Follow-up for BCR
Alnachoukati 2018	Retrospective	Vanguard XP total knee system (Zimmer Biomet)	146	2	1 for arthrofibrosis / cyclops lesion; 1 for tibial‑tray subsidence (under sizing)	Mean: 12 months
Barberis 2024	Retrospective	Journey II XR (Smith & Nephew, Memphis, TN, USA)	102	2	1 for ACL tear → converted to CR insert; 1 for arthrofibrosis + synovectomy	Mean: 32.4 months
Baumann 2018	Prospective	Vanguard XP total knee system (Zimmer Biomet)	34	1	1 for Arthroscopic debridement for stiffness	Mean: 18 months
Eggenberger 2022	Retrospective	Vanguard XP total knee system (Zimmer Biomet)	195	22	19 for aseptic tibial loosening; 1 for infection; 1 for instability with PCL rupture; 1 for arthrofibrosis	Mean: 60 months
Kalaai 2021	Retrospective	Did not specify	61	1	1 for Valgus thrust	Mean: 33.6 months
Pelt 2019	Retrospective	Vanguard XP total knee system (Zimmer Biomet)	141	19	5 for isolated tibial loosening; 4 for pain; 3 for ACL impingement; 3 for unknown; 2 for femoral + tibial loosening; 1 for ACL deficiency; 1 for arthrofibrosis	Mean: 36 months
Inui 2023	Retrospective	Journey II XR (Smith & Nephew, Memphis, TN, USA)	50	0	n/a	Mean: 24 months
Christensen 2017	Retrospective	Vanguard XP total knee system (Zimmer Biomet)	65	3	1 for septic failure; 1 for ACL impingement (aseptic); 1 for metal‑allergy / tibial loosening (aseptic)	Mean: 18 months
Tria 2021	Retrospective	Journey II XR (Smith & Nephew, Memphis, TN, USA)	47	2	1 for recurrent patellar subluxation; 1 for stiffness	Mean: 29.6 months
Troelsen 2020	Randomized Controlled Trial	Vanguard XP total knee system (Zimmer Biomet)	24	1	1 for tibial‑island fracture	Mean: 24 months
Lavoie 2023	Randomized Controlled Trial	Hermes 2 C (Ceraver-Osteal, Roissy, France)	39	2	1 for Stiffness; 1 for aseptic tibial loosening	Mean: 60 months
Singh 2023	Retrospective	Journey II XR (Smith & Nephew, Memphis, TN, USA)	133	6	2 for infection; 2 for arthrofibrosis; 1 for ACL rupture; 1 for stiffness (liner exchange)	Mean: 28.2 months
West 2019	Prospective	Journey II XR (Smith & Nephew, Memphis, TN, USA)	50	1	1 for stiffness	Minimum: 1-year

Abbreviations: ACL = Anterior Cruciate Ligament; BCR = Bicruciate-Retaining; CR = Cruciate-Retaining; PCL = Posterior Cruciate Ligament; USA = United States of America; TN = Tennessee; n/a = not applicable; No. = number.

### Revision incidence

**Fig. 2:**
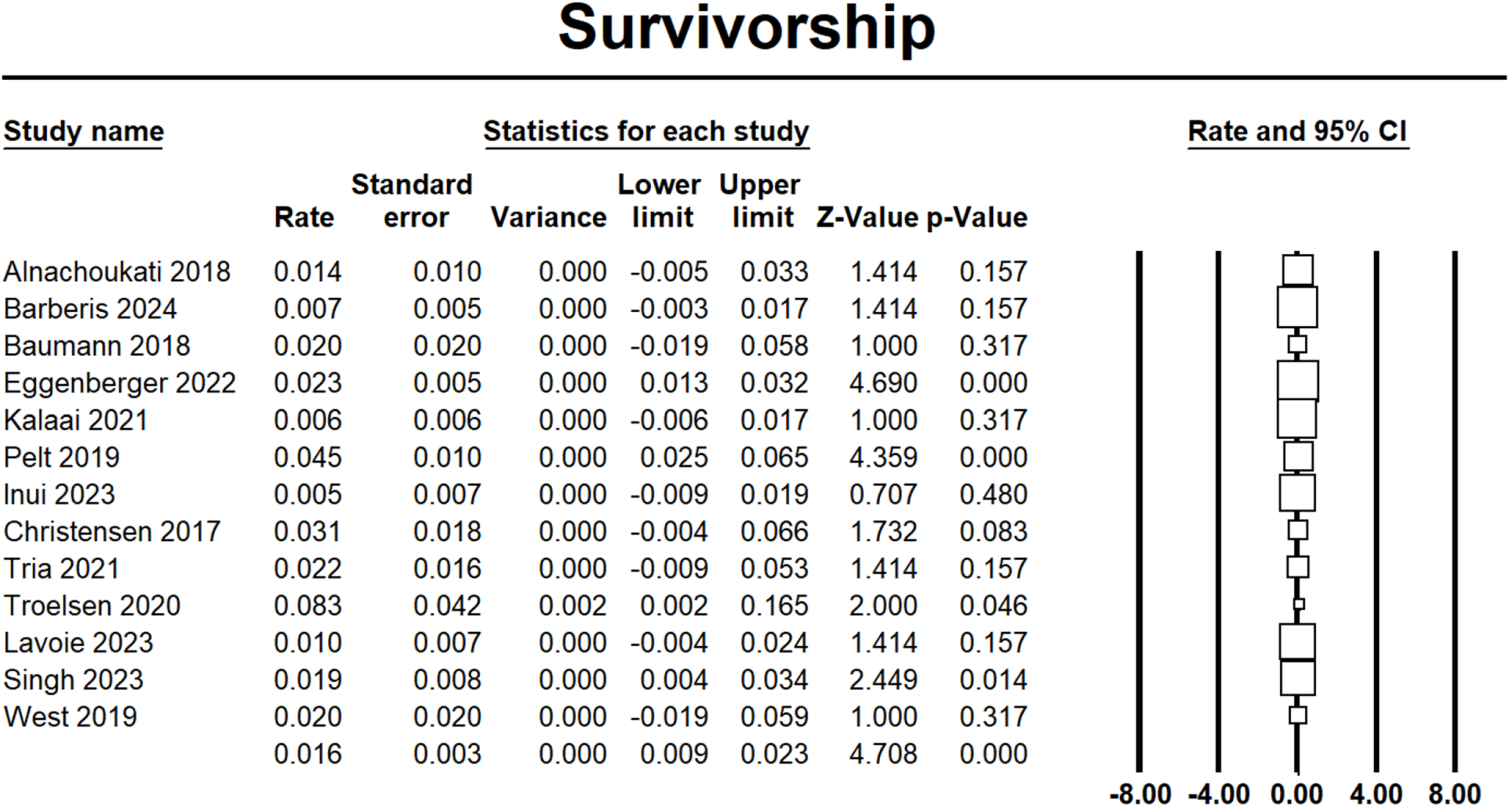
Pooled analysis for all-cause revision among BCR TKA

Across all studies 62 revisions were reported, equivalent to 5.7% of implanted knees. When pooled as events-per-person-year, the all-cause revision incidence was 1.6 per 100 person-years (95% CI 0.9–2.3). Statistical heterogeneity was substantial (I² = 75.5%, *p* < 0.001) (Fig. 2).

### Failure modes

Aseptic tibial loosening/subsidence 29/62 (47%), stiffness/arthrofibrosis/cyclops lesion 11/62 (18%), instability or ligament/patellofemoral-related failure 10/62 (16%), infection/septic failure 4/62 (6%), pain 4/62 (6%), unknown 3/62 (5%), and tibial-island fracture 1/62 (2%).

## Discussion

This systematic review synthesized data from 14 studies encompassing 1,087 s-generation BCR TKAs, with a mean follow-up of 2.6 years. The pooled all-cause revision incidence was 1.6 per 100 person-years (95% CI 0.9–2.3), with a total revision rate of 5.7%. However, significant heterogeneity was observed across studies (I² = 75.5%, *p* < 0.001). Aseptic tibial loosening was the most common mode of failure, accounting for 47% of revisions, followed by stiffness/arthrofibrosis (18%). These findings highlight the higher survivorship observed for the 2nd generation BCR TKA implants, encouraging broader adoption.

Historically, first-generation BCR TKAs were notorious for their high failure rates. In a 22-year follow-up, Sabouret and colleagues reported only 83% survivorship and noted tibial loosening as the main cause of failure [21]. Modern implants may have improved surgical outcomes. Cohorts using Journey II XR and Vanguard XP now show 96–98% component survival at 5–7 year review [6, 7, 12]. Barberis et al. followed 102 Journey II XR knees and found 98% revision‑free survival at 24 months, while Singh et al. reported 97.3% aseptic revision‑free survival at a mean 2.3 years in 133 Journey II XR knees [11, 19]. Mid-term signals from contemporary cohorts and randomized studies are encouraging, but device-specific variability remains. Our pooled 1.6 revisions per 100 person-years therefore reflects strong early- to mid-term survivorship for second-generation BCR systems while underscoring the need for longer follow-up and continued post-market surveillance across implant families.

Failure patterns have evolved markedly since the first BCR designs. First-generation implants introduced in the 1970–1980 s showed revision rates approaching 15% within the first decade, and multiple historical reviews showed that aseptic tibial loosening was the dominant cause of failure (3, 4). The improvement in implant survivorship is attributed to a unified set of design upgrades. Modern BCR systems pair high-porosity titanium or tantalum coatings that accelerate osseointegration with anatomically contoured femoral components that distribute load more physiologically and side-specific polyethylene inserts that maintain ACL clearance. When placed with patient-specific guides, computer navigation, or robotics—tools that refine tibial sizing, coronal alignment, and ligament balance— these features restore more physiologic rollback and internal rotation while addressing the fixation and polyethylene-wear problems that limited first-generation designs [6, 7, 12]. These innovations appear to have reduced fixation-related failures, and, in turn, the overall revision incidence observed for second-generation BCR implants.

These technological advances translate into practical guidance for surgeons. Patients who have a functionally intact ACL, neutral or correctable alignment, and adequate bone stock are the best candidates for a BCR TKA [13]. To realize that benefit, surgeons must ensure accurate tibial sizing, neutral mechanical alignment, and intra-operative verification that the ACL clears the polyethylene insert; failure on any of these points predisposes to late loosening or early soft-tissue impingement [22]. When those conditions are met, preserving both cruciates appears to deliver a knee that feels closer to normal. In 146-knee series, 91% of BCR TKA recipients said their knee “always or sometimes feels normal” and 94% were at least neutrally satisfied [10]. Likewise, in a staged bilateral study, 89% of patients preferred the BCR TKA over a contralateral posterior‑stabilized implant [23]. Together, these clinical observations suggest that the kinematic and proprioceptive advantages of ligament preservation are perceivable to most patients—and can be achieved without compromising the long-term implant survivorship.

This study has several limitations. The inclusion of mainly retrospective studies may introduce data collection bias. Furthermore, the high heterogeneity found among analyzed studies should be considered when interpreting the pooled revision estimate. Although we accounted for the between-study variability using a random-effects model, differences in implant design, preoperative care, and follow-up methods may still limit the generalizability of the pooled findings. In addition, the heterogeneity limits our ability to determine whether any single factor was primarily responsible for the variability in the studies. This heterogeneity may also influence the distribution of failure modes, as the contribution of aseptic tibial loosening, infection, instability, and arthrofibrosis may vary across implant systems and follow-up periods. Also, various implant designs and manufacturers were included, which may bias our results. However, our study provides a comprehensive evaluation of the modern BCR TKA implant survivorship. Nonetheless, there remains a need for studies evaluating clinically meaningful improvements in PROMs in patients who underwent primary TKA using BCR designs. The results of this study highlight the improved survivorship with second generation BCR TKA, encouraging their adoption, although these findings must be interpreted in the context of the observed heterogeneity.

## Conclusions

Second-generation BCR TKA demonstrates improved early-to-midterm revision rate, with an all-cause revision incidence of 1.6 per 100 person-years in this meta-analysis. Aseptic tibial loosening remained the most prevalent failure mode, followed by stiffness/arthrofibrosis. The distinct failure profile—particularly the predominance of aseptic loosening—warrants further investigation into implant design, surgical technique, and patient selection to optimize outcomes. While promising in theory for restoring native knee kinematics, there is a need for prospective data analyzing the long-term survivorship and clinically meaningful improvements in patient-reported outcomes.

## Supplementary Information

Below is the link to the electronic supplementary material.

Supplementary Material 1

## Data Availability

No datasets were generated or analysed during the current study.
