# Supplementary material for: Contemporary bicruciate-retaining total knee arthroplasty implants demonstrate favorable survivorship: a systematic review and meta-analysis of 1576 knees
Source: Eur J Orthop Surg Traumatol. 2026 Jul 1;36(1):268. doi: 10.1007/s00590-026-04811-0 (PMC13323789; doi:10.1007/s00590-026-04811-0)
Supplement: Supplementary file 1 — Supplementary Material 1 [file 590_2026_4811_MOESM1_ESM.docx]

**Appendix A. Newcastle–Ottawa Scale (maximum = 9) for the 14 included studies**

| **Study ID** | **Study design** | **Selection (4 pts)** | **Comparability (2 pts)** | **Outcome / Exposure (3 pts)** | **Total(/9)** | **Quality*** |
| --- | --- | --- | --- | --- | --- | --- |
| Alnachoukati 2018 | Retro cohort | 3 | 0 | 3 | 6 | Moderate |
| Barberis 2024 | Retro cohort | 3 | 0 | 3 | 6 | Moderate |
| Baumann 2018 | Pro cohort | 4 | 1 | 3 | 8 | High |
| Eggenberger 2022 | Retro cohort | 4 | 1 | 3 | 8 | High |
| Kalaai 2021 | Retro cohort | 4 | 1 | 3 | 8 | High |
| Pelt 2019 | Retro cohort | 3 | 0 | 3 | 6 | Moderate |
| Inui 2023 | Retro cohort | 4 | 1 | 3 | 8 | High |
| Christensen 2017 | Retro cohort | 4 | 0 | 3 | 7 | High |
| Tria 2021 | Retro cohort | 3 | 0 | 3 | 6 | Moderate |
| Singh 2023 | Retro Cohort | 3 | 0 | 2 | 5 | Moderate |
| West 2019 | Pro cohort | 3 | 0 | 3 | 6 | Moderate |
|  |  |  |  |  |  |  |
|  |  |  |  |  |  |  |

*High = 7–9; Moderate = 5–6; Low ≤ 4.

**Appendix B. Cochrane Risk-of-Bias 2 (parallel-group) assessment for the two included randomized controlled trials**

| **Study** | **Rnd. process** | **Dev. from int.** | **Missing data** | **Outcome meas.** | **Select. report** | **Overall** |
| --- | --- | --- | --- | --- | --- | --- |
| **Troelsen 2020** | **Low** | Some concerns | **Low** | **Low** | Some concerns | **Some concerns** |
| **Lavoie 2023** | Some concerns | Some concerns | **High** | **Low** | Some concerns | **High** |

**Search Strategy**

Ovid MEDLINE(R) ALL <1946 to January 03, 2025>

1 Arthroplasty, Replacement, Knee/ 34838

2 ((arthroplast* or replac*) and (knee or knees)).mp. 59599

3 (TKA or TKR).tw. 21216

4 1 or 2 or 3 60090

5 (bi-cruciate or bicruciate).mp. 295

6 (BCS or BCL).tw. 96627

7 5 or 6 96856

8 4 and 7 241

9 limit 8 to yr="2010 -Current" 222

Embase <1974 to 2025 January 03>

1 exp knee replacement/ 30080

2 ((arthroplast* or replac*) and (knee or knees)).mp. 81934

3 (TKA or TKR).tw. 25064

4 1 or 2 or 3 82835

5 (bi-cruciate or bicruciate).mp. 348

6 (BCS or BCL).tw. 127744

7 5 or 6 128023

8 4 and 7 314

9 limit 8 to yr="2010 -Current" 286

Cochrane Library CENTRAL

Date Run: 06/01/2025 20:13:00

Comment:

ID Search Hits

#1 MeSH descriptor: [Arthroplasty, Replacement, Knee] this term only 4125

#2 (arthroplast* or replac*) and (knee or knees) 12962

#3 (TKA or TKR):ti OR (TKA or TKR):ab 5093

#4 #1 OR #2 OR #3 13155

#5 bi-cruciate or bicruciate 42

#6 (BCS or BCL):ti OR (BCS or BCL):ab 2091

#7 #5 OR #6 2120

#8 #4 AND #7 with Cochrane Library publication date Between Jan 2010 and Jan 2025 51

Scopus

( TITLE-ABS-KEY ( ( ( arthroplast* OR replac* ) AND ( knee OR knees ) ) ) OR TITLE ( tka OR tkr ) OR ABS ( tka OR tkr ) ) AND ( TITLE-ABS-KEY ( bi-cruciate OR bicruciate ) OR TITLE ( bcs OR bcl ) OR ABS ( bcs OR bcl ) ) AND PUBYEAR > 2009 267

Web of Science Core Collection

1. ((ALL=(( arthroplast* OR replac* ) AND ( knee OR knees ))) OR TI=(tka OR tkr)) OR AB=(tka OR tkr) 69,454

2. ((ALL=(bi-cruciate or bicruciate) OR TI=(BCS or BCL)) OR AB=(BCS or BCL)) 113,831

3. #2 AND #1 245

4. #2 AND #1 and 2025 or 2024 or 2023 or 2022 or 2021 or 2020 or 2019 or 2018 or 2017 or 2016 or 2015 or 2014 or 2013 or 2012 or 2011 or 2010 (Publication Years) 220
